# Retroviral Antisense Transcripts and Genes: 33 Years after First Predicted, a Silent Retroviral Revolution?

**DOI:** 10.3390/v13112221

**Published:** 2021-11-04

**Authors:** Roger H. Miller, Alexis Zimmer, Gilles Moutot, Jean-Michel Mesnard, Nathalie Chazal

**Affiliations:** 1 CyberGenomics LLC, Brookeville, MD 20833, USA; 2 DHVS—Département d’Histoire des Sciences de la Vie et de la Santé, Faculté de Médecine, Université de Strasbourg, 4 Rue Kirschleger, CEDEX, F-67085 Strasbourg, France; alexis.zimmer@unistra.fr; 3 Centre d’Etudes Politiques et Sociales (CEPEL), Département de Sciences Humaines et Sociales, Université de Montpellier, 34090 Montpellier, France; gilles.moutot@umontpellier.fr; 4 Institut de Recherche en Infectiologie de Montpellier (IRIM), Université de Montpellier, CNRS, 1919 Route de Mende, CEDEX 5, 34293 Montpellier, France; jean-michel.mesnard@irim.cnrs.fr

**Keywords:** virology, retrovirology, oncogenesis, antisense transcript, antisense protein, HTLV-1, HIV-1, ASP

## Abstract

Paradigm shifts throughout the history of microbiology have typically been ignored, or met with skepticism and resistance, by the scientific community. This has been especially true in the field of virology, where the discovery of a “*contagium vivum fluidum*”, or infectious fluid remaining after excluding bacteria by filtration, was initially ignored because it did not coincide with the established view of microorganisms. Subsequent studies on such infectious agents, eventually termed “viruses”, were met with skepticism. However, after an abundance of proof accumulated, viruses were eventually acknowledged as defined microbiological entities. Next, the proposed role of viruses in oncogenesis in animals was disputed, as was the unique mechanism of genome replication by reverse transcription of RNA by the retroviruses. This same pattern of skepticism holds true for the prediction of the existence of retroviral “antisense” transcripts and genes. From the time of their discovery, it was thought that retroviruses encoded proteins on only one strand of proviral DNA. However, in 1988, it was predicted that human immunodeficiency virus type 1 (HIV-1), and other retroviruses, express an antisense protein encoded on the DNA strand opposite that encoding the known viral proteins. Confirmation came quickly with the characterization of the antisense protein, HBZ, of the human T-cell leukemia virus type 1 (HTLV-1), and the finding that both the protein and its antisense mRNA transcript play key roles in viral replication and pathogenesis. However, acceptance of the existence, and potential importance, of a corresponding antisense transcript and protein (ASP) in HIV-1 infection and pathogenesis has lagged, despite gradually accumulating theoretical and experimental evidence. The most striking theoretical evidence is the finding that *asp* is highly conserved in group M viruses and correlates exclusively with subtypes, or clades, responsible for the AIDS pandemic. This review outlines the history of the major shifts in thought pertaining to the nature and characteristics of viruses, and in particular retroviruses, and details the development of the hypothesis that retroviral antisense transcripts and genes exist. We conclude that there is a need to accelerate studies on ASP, and its transcript(s), with the view that both may be important, and overlooked, targets in anti-HIV therapeutic and vaccine strategies.

## 1. Introduction

Virology is a very recent scientific discipline, with the concept of viruses dating back barely more than a century. This explains, at least in part, why these biological agents have remained virtually absent from the philosophy of biology until recently, as pointed out by Thomas Pradeu and colleagues [1]. However, other reasons can explain this gap, most notably the unparalleled diversity and complexity of viruses, which make them difficult biological entities to define and categorize. In fact, viruses are the most abundant biological entities on Earth (estimated 1.0 × 10^31^ individual viruses) [2,3,4], capable of infecting every type of living organism and exhibiting a diversity greater than the cumulative diversity of organisms in the three kingdoms of life [3,5]. In addition, the very concept of viruses had great difficulty in gaining acceptance in the era of bacteriology born of the work of Louis Pasteur and Robert Koch. Later on, the unique nature of viruses raised major conceptual questions (e.g., the origin of life, definition of living organisms, etc.) and the accelerated pace of discoveries provoked numerous paradigm shifts, which also contributed to this delay. We show here how virology, and more particularly retrovirology, has progressed rapidly in the past century. We focus particularly on retroviral antisense genes that question, and modify, scientific preconceptions on genetics.

## 2. Virology: The Birth of a Discipline

Virology is a discipline born from a change in the perception of the world of microorganisms. On 30 April 1878, Louis Pasteur defended “The theory of germs and its applications to medicine and surgery” (a theory also supported by Joseph Lister and Robert Koch) [6]. The representation of microorganisms as solely bacteria was quickly called into question. In 1892, without realizing the real significance of his discovery, Dimitry Ivanovski, a Russian botanist, discovered the tobacco mosaic virus (TMV) while working on a disease affecting tobacco plants using a Chamberland filter. This filter, developed in 1886 by Pasteur’s collaborator Charles Chamberland during the typhoid fever epidemic in Paris, retained bacteria while permitting liquid to pass through. Ivanovski demonstrated that the “agent” present in the liquid sap of diseased tobacco plants transmitting the disease was not retained by the Chamberland filter (i.e., was not a bacteria), and that the filtrate obtained was “infectious” [7]. In 1898, Martinus Beijerinck, a Dutch microbiologist, reproduced Ivanovski’s experiment [8]. He then used the word “*virus*”, a term mentioned in old medical textbooks to designate a substance capable of transmitting a disease [9,10], to define a noncorporeal living fluid, the “*contagium vivum fluidum*”. The combined work of Ivanovski and Beijerinck, while clearly establishing the existence of TMV, did not attract the attention of the scientific community, and eventually provoked skepticism, as it upset the established bacterial model. Thus, in 1903, Emile Roux, a member of the Pasteur Institute, challenged this hypothesis: “The conception of this *contagium vivum fluidum* is very original, but before admitting it, could we not suppose that a very fine microbe, provided with spores, has penetrated the depth of the agar?” [11]. This episode illustrates the great difficulty the scientific community had in accepting the existence of viruses, and illustrates a tendency often repeated in science when confronting an idea outside of the norm.

Subsequently, many other viruses were identified by this approach: the animal virus responsible for foot-and-mouth disease (FMDV) [12], followed by the discovery of the first virus known to be pathogenic to humans, yellow fever virus (YFV) [13]. In Denmark, in 1908, a physician, Vilhelm Ellermann, and a veterinarian, Oluf Bang, showed that leukemia in chickens was caused by the avian leukosis virus (ALV). In 1910, Peyton Rous prepared an extract from samples prepared from a tumor obtained from a chicken. After passage through a Chamberland filter, the extract was injected into healthy chickens and produced new tumors. From this experiment, Rous concluded that the cancer had been caused by a virus, now known as Rous sarcoma virus (RSV) [14]. During the First World War, Frederick Twort and Félix D’Herelle independently discovered viruses that infect bacteria, termed bacteriophages [15,16]. Then came the discovery of the rabies, vaccinia, poliomyelitis, and human influenza viruses [17]. As the list of new viruses was growing, a new technical advance, the use of electron microscopy, permitted direct viewing of viruses. In 1939, TMV, the virus involved in the establishment of the theory of viruses, was the first virus observed using electron microscopy [18]. The TMV was subsequently used as a model for structural and biochemical studies [19], while the study of bacteriophages made it possible to define the main stages of the replication cycle [20,21,22]. Next, the development of cell culture techniques, with the culturing of the poliomyelitis virus in 1949 [23] proved to be a decisive turning point for the study of viruses, facilitating both the identification of new viruses as well as the production of vaccines. In 1957, André Lwoff proposed a new definition of viruses: “Viruses are infectious and potentially pathogenic; they are nucleoprotein entities possessing a single type of nucleic acid (RNA or DNA); they are reproduced (by the cell) from their genetic material; they are unable to grow and divide; they lack the Lipmann system” [24]. Then, in 1966, following the discovery of ribosomes, a new element was added to this definition: viruses use the translation mechanisms of their host cells [25]. Thus, in slightly less than 75 years after their discovery, the fundamental nature of viruses was delineated.

## 3. Retrovirology: A Cornucopia for Understanding Oncogenesis

Retroviruses have been an important focus of research for many laboratories since the beginning of virology. The first description of a disease linked to a retrovirus, the equine infectious anemia virus (EIAV), was made by the French veterinarians Henri Vallée and Henri Carré in 1904 [26]. To study the mode of transmission, Vallée and Carré filtered sera from sick horses and showed that the filtrate could transmit anemia to healthy horses. Shortly thereafter, as discussed above, Ellermann and Bang showed that leukemia in chickens was caused by a virus [27], and Rous demonstrated that the sarcoma observed in chickens was also induced by a virus [14]. Surprisingly, the first demonstrations linking a virus and an oncogenic mechanism had no immediate impact in the field of oncology. During the following decades, many avian retroviruses, as well as those infecting mammals (e.g., mouse, cats, cattle, monkeys), involved in neoplastic diseases were discovered [28,29,30], and the investigation of retroviruses lead to major advances in biology.

Among the great scientific advances in biology in the 20th century, the discovery of reverse transcriptase was a major step in understanding the mechanism of retrovirus replication. This finding also played a key role in elucidating certain processes in viral oncogenesis, the discovery of HIV-1, and the development of small molecule anti-HIV-1 therapeutics, and contributed to a revolution in molecular biological techniques. The discovery of reverse transcriptase was carried out independently in the laboratories of Howard Temin (model: RSV) and David Baltimore (model: mouse leukemia virus, MLV). In 1975, the importance of their discovery was acknowledged by the award of the Nobel Prize in Physiology or Medicine shared with Renato Dulbecco. The discovery process began in the 1960s, at a time when molecular tools were not very well developed and the mechanisms of replication of viruses were derived almost exclusively from observations made using various inhibitors. Thus, retroviral replication was shown to be sensitive to inhibitors of both DNA and RNA synthesis [31,32]. In 1964, Temin proposed the existence of a DNA provirus and its integration into the cellular genome [33]. Unfortunately, these experiments did not convince the scientific community. Subsequently, Temin proposed that the enzyme required for RSV DNA pre-existed in virions and could act upon entry of the virus into the cell. Later, Satoshi Mizutani, a postdoctoral fellow in Temin’s laboratory, definitively demonstrated the presence of a viral enzyme within virions capable of inducing DNA synthesis from viral RNA. When Temin presented his results for the first time in 1970, the finding was met with skepticism, and his presentation was strongly attacked. Subsequently, Temin received a call from David Baltimore announcing his discovery of an enzyme with the same characteristics in MLV virions. Two papers were published back-to-back in the 27 June 1970 issue of Nature [34,35], along with a commentary summarizing the findings entitled: “Central Dogma Reversed”. Thus, it took more than 10 years for Temin to convince his peers, who sometimes called his hypothesis heretical, of the validity of his work. The acceptance of the existence of reverse transcriptase by the scientific community was then quite rapid and undoubtedly aided by the fact that definitive experiments were published by these two independent groups, and then confirmed by a third team a few weeks later [36], leaving little room for skepticism.

Shortly after this major breakthrough, further significant advances were made in understanding the molecular mechanisms of retroviral carcinogenesis. In 1970, Steve Martin, while isolating a heat-sensitive mutant of RSV, was unable to transform cells infected at nonpermissive temperatures, but at a temperature that permitted cells to replicate normally. This was a seminal observation that led to the hypothesis that a viral gene was required for cell transformation, but not for replication [37]. The same year, the work of Duesberg and Vogt suggested, by studying ALV and RSV viruses without transforming activity, the presence of a viral sequence in RSV responsible for the transformation of fibroblasts [38]. This gene, named *src* (encoding the sarcoma protein), termed an oncogene because of its transforming capacity, was mapped to the 3’ end of the viral genome [39]. Dominique Stéhelin, Michael Bishop, and Harold Varmus then investigated the origin of this oncogene that was unnecessary for the replication of the virus. Using differential hybridization approaches led them to identify, in the genome of normal uninfected chicken cells, the presence of a DNA sequence with homology to *src*. Next, they demonstrated that the ability of RSV to induce tumors resulted from the acquisition of this sequence captured from the cellular genome during the replication cycle of the retrovirus. Thus, the discovery of the first cellular oncogene, called *c-src,* opened a new avenue for understanding the molecular mechanisms leading to carcinogenesis. Later, Vogt and Duesberg, published in 1977 and 1979, respectively, worked on the avian acute leukemia viruses MC29 and avian erythroblastosis virus, and discovered new viral oncogenes derived from cellular oncogenes, which are known today as *myc* and *erb* [40,41]. In addition, the *ras* oncogene was identified in a murine virus [42]. Since the pioneering discovery of the first oncogene, originating from a cellular proto-oncogene, in a chicken virus, which earned Bishop and Varmus the Nobel Prize in Physiology or Medicine in 1989, oncogene research has become a central focus in human cancer genetics. Overall, several oncogenes originally identified in retroviruses are now recognized as major drivers of human cancer.

## 4. Discovery of the First Human Retrovirus: HTLV-1, Birth of Human Retrovirology

As mentioned, animal retroviruses were the first oncogenic viruses identified. These retroviruses cause carcinomas, lymphomas, sarcomas, etc. In the 1970s, when Japanese researchers were first describing adult T-cell leukemia (ATL) [43] Robert Gallo’s group discovered cytokine 2, now called interleukin-2 (IL-2), which promotes T-cell growth. This breakthrough, along with the development of sensitive and specific techniques for testing for reverse transcriptase activity, was useful for the long-term culture of human T-cells isolated from leukemia patients (ATL). Subsequently, this aided the discovery of the first human retrovirus, human T-cell leukemia virus-1 (HTLV-1) [44]. The study of this virus has opened new perspectives in retrovirology. It was shown that, like all retroviruses, the HTLV-1 provirus sequence contains a promoter in its 5’ long terminal repeat (5′LTR) and ORFs encoding structural proteins (Gag, Env) and proteins with enzymatic activity (Pol, Pro). In the 3’ end of its genome, the virus also possesses several ORFs encoding regulatory (i.e., Rex and Tax) or auxiliary (i.e., p12, p13, and p30) proteins translated from singly or doubly spliced mRNAs. This discovery changed the vision of retroviruses by demonstrating that not only those retroviruses can infect humans, but also that retroviruses can be pathogenic for humans. Shortly afterwards, the team of G. de Thé demonstrated that HTLV-1 infection, in addition to causing leukemia, was also associated with a chronic neurological disease: tropical spastic paraparesis, later also called HTLV-1-associated myelopathy (TSP/HAM) [45]. The work on HTLV-1 and its role in oncogenesis gradually led to the proposition that the Tax protein, essential for the expression of proviral genes from the 5′LTR, could also play a central role in T-cell transformation. Subsequently, for more than 35 years, virtually all efforts were directed towards the study of the viral transactivator protein, Tax, which was found to immortalize human lymphocytes in vitro and to transform them in vivo in transgenic animal models. Numerous studies have demonstrated that Tax activates CREB, NF-κB, and SRF signaling pathways [46], and causes numerous effects on host cell proteins involved in cell cycle regulation, cell division, tumor suppression, and DNA repair [47,48,49,50,51,52]. However, as we shall see below, Tax was not the sole actor on this stage.

## 5. Antisense Transcription and Antisense Proteins in Retroviruses: A Significant Hypothesis

Transcription is a major step in the process of gene expression. In the cell, most transcription occurs using the DNA strand of negative polarity (−) that contains gene sequences. The resulting “sense” mRNA is translated into protein(s). Transcription of the complementary DNA strand of positive polarity (+), into “antisense” mRNA, was not considered, or even envisaged, for many years. In 1972, the hypothesis of antisense transcription at the origin of natural untranslated antisense RNAs was postulated [53]. Some double-stranded DNA viruses are known to make use of antisense transcription, notably the *Herpesviridae* [54]. These viruses are characterized, in particular, by their ability to induce persistent infections, with lytic and latent phases. A great deal of work has been done to decipher the molecular mechanisms leading to viral latency and reactivation, and has led to the demonstration that the products of sense and antisense transcription allow the passage from the lytic cycle to a latent cycle. However, antisense transcripts were unknown for RNA viruses, or double-stranded DNA viruses that replicate via an RNA intermediate (e.g., Hepadnaviruses). Retroviruses, an example of RNA viruses, share a common genomic organization in which the 5′LTR is followed by the *gag*, *pol* (*pro*, *pol*), and *env* genes, and ends with the 3′LTR. Often overlapping regulatory and accessory genes are also present in the 3′ region of the genome. The original assumption was that all retroviral proteins were translated from sense mRNA. However, in 1988, 5 years after the discovery of HIV-1 and more than 80 years after the discovery of the first retrovirus, a paradigm shift took place in the world of virology with the prediction that retroviruses also express proteins translated from antisense mRNA [55].

## 6. The 10th Gene of HIV-1 Encodes a Protein Expressed from Antisense mRNA: A Shift in Perspective

### 6.1. Predicting the Existence of an HIV-1 Antisense Protein

Early work in genetics [56] led to the hypothesis of “one gene-one enzyme” for the expression of proteins [57]. At that time, double-stranded DNA was thought to consist of coding and noncoding strands. Overlapping genes were unknown, and proteins were thought to be encoded only on one strand of DNA via sense polarity mRNA. This understanding of gene expression was thought to represent the simplicity of genetic evolution. However, subsequent investigation revealed that genome organization and gene expression were much more complex. One such example is the prediction, and subsequent confirmation, of the existence of a protein encoded by the plus strand DNA of HIV-1 via an antisense mRNA transcript [55]. The ORF of ASP overlaps, and is complementary to, the HIV envelope gene (*env*) and the Rev response element (RRE) located within *env*. This represents an example of proteins translated from overlapping and complementary mRNA transcripts. The following is an overview of the development of the hypothesis for the presence of retroviral antisense proteins.

### 6.2. Lessons from the Hepadnavirus Genome

**Figure 1:**
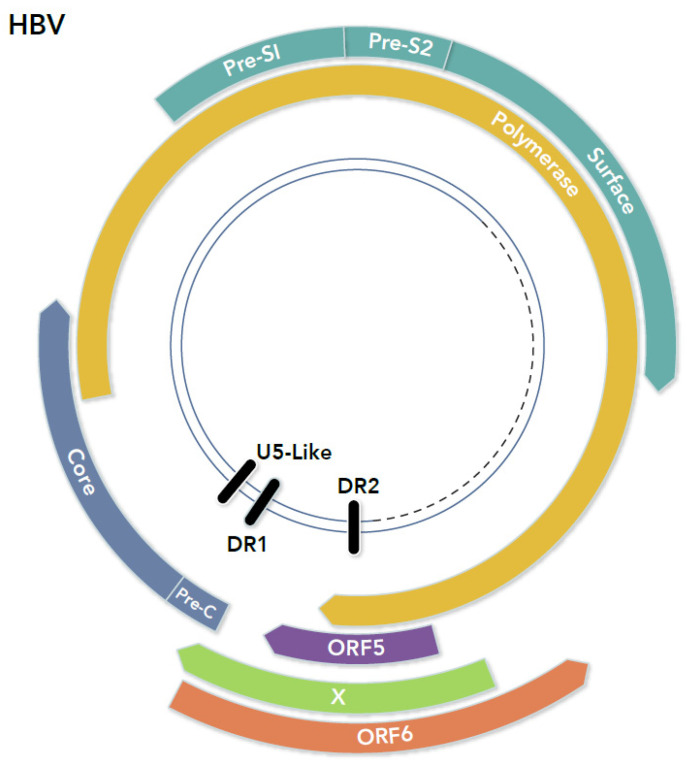
Open reading frames in the HBV genome. The color scheme in all figures is: for ORFs or proteins, antisense ORF (red), replicase (orange), capsid (blue), envelope (aquamarine), and regulatory (green); and for nucleotide elements (black).

Hepadnaviruses are a family of small DNA viruses, infecting both mammals and birds, that possess an extremely compact genome organization [58]. While these viruses occasionally integrate into the host genome, integration is not requisite for their replication or gene expression. The genome of the prototypic member of the family, hepatitis B virus (HBV), is a circular, partially double-stranded molecule of approximately 3200 bp (Figure 1). Optimal use of its genome is accomplished by several methods. First, there are no noncoding regions. Thus, all cis-acting regulatory elements are located within genes. These elements include short direct-repeated (DR) sequences used in virus replication, a poly-A addition sequence, an enhancer of transcription, promoters, and other elements. Second, HBV encodes proteins in overlapping translation frames. For example, the virus presurface (Pre-S) and surface proteins are encoded entirely within the polymerase gene sequence in an alternate translation frame. In addition, both the core and X protein genes partially overlap the polymerase gene. Thus, HBV utilizes its small genome in a very efficient manner.

Original sequence analysis of the HBV genome revealed the presence of eight ORFs greater than 100 codons [59]. Four ORFs were located on what was termed the “L” strand and four on the “S” strand of DNA. Further investigation revealed that the S strand corresponded to the minus DNA strand of the virus genome and the ORFs shown to encode the core (nucleocapsid), surface (envelope), polymerase (replicase), and X (i.e., unknown function) proteins. Once additional complete genome sequences for hepadnaviruses were available, an analysis was performed searching for additional conserved ORFs on both DNA strands. Two ORFs, termed ORFs 5 and 6, were found conserved in the genomes of all HBV and animal hepadnaviruses examined (Figure 1) [58,60]. ORF5, located on the minus DNA strand, is contained entirely within the coding sequence of the X protein in a different translation frame. Thus, it could be expressed by any of the known HBV mRNAs. However, no initiation codon is present, and no evidence has been reported for its expression. ORF6, located on the plus strand of the HBV genome, would need to be translated via an antisense transcript. A protein of approximately 200 amino acids could be produced if translation begins from the first initiation codon. ORF6 overlaps the pre-core, X, and polymerase ORFs. This ORF was found to be present in all 18 full-length HBV genomes examined, as well as in the genomes of duck hepatitis B virus, ground squirrel hepatitis virus, and woodchuck hepatitis (WHV). Thus, since ORF6 appeared to be conserved in the genomes of all hepadnaviruses studied, it seemed possible, or even likely, that the genome of a common ancestral virus encoded a functional antisense protein.

Since hepadnaviruses package double-stranded DNA genomes, and retroviruses package RNA genomes in virions, it was unexpected that they shared many similarities in genome organization, mechanism of replication, or gene expression. However, this proved not to be the case, and many similarities were eventually revealed. First, both were shown to replicate using reverse transcription of an RNA intermediate [61,62]. Second, a U5-like regulatory sequence from retrovirus LTRs was found in the terminal repeats of the linear hepadnavirus RNA intermediate. Third, there was amino acid sequence homology between the nucleocapsid proteins of members of both families. Finally, the organization of the linear RNA intermediate of hepadnaviruses mimicked the *gag-pol-env-regulatory protein(s)* pattern of the RNA genome of retroviruses. The discovery of these similarities led to the hypothesis that hepadnaviruses and retroviruses shared a common ancestor in their evolution [63].

### 6.3. Formulation of the Hypothesis for an HIV-1 Antisense Protein

**Figure 2:**
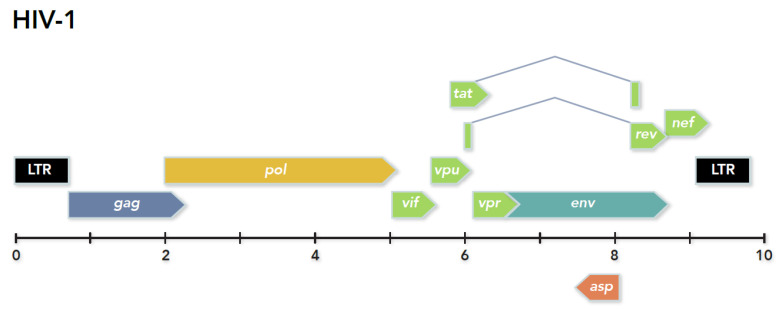
Organization of the HIV-1 Group M genome.

Since hepadnaviruses and retroviruses likely share a common ancestor, it followed that at least some retroviruses could express an antisense protein. Thus, an examination of all retrovirus full-length genome sequences available in 1987 was undertaken to search for antisense ORFs of a significant size. Analysis revealed the presence of antisense ORFs in the 3′ genome region of HIV-1 and -2, simian immunodeficiency virus (SIV), HTLV-1 and HTLV-2, bovine leukemia virus (BLV), simian T cell leukemia virus (STLV), Visna virus, and EIAV [55,64]. Many, but not all, of the ORFs contained initiation codons, implying a protein could be translated from antisense mRNA. Of all retroviruses viruses studied, the strongest theoretical case could be made for an HIV-1 antisense protein (ASP) due to conservation of the ORF in the relatively large number of complete genome sequences available at that time. Specifically, an antisense ORF of approximately 190 amino acids, beginning with the first initiation codon, was identified opposite to the *env* gene in all 12 complete HIV-1 genome sequences in GenBank (Figure 2). The ORF was also present in an additional 12/13 *env* sequences available (i.e., incomplete genome sequences). Certainly, it could be argued that the ORF was conserved due to the constraints imposed by complementary overlapping *env* and RRE sequences. However, evidence for the expression of an antisense protein was compelling, since the signal sequences necessary for production of an antisense mRNA transcript (i.e., promoter, poly-A addition sequence, etc.), and translation of the protein (i.e., canonical protein start sequence, etc.), were both present and conserved in all nucleotide sequences analyzed [55]. These data provided convincing theoretical evidence for production of both an mRNA antisense transcript and translated protein during HIV-1 replication. As a result of the publication of this hypothesis, additional corroborating theoretical evidence followed suggesting that HIV-1 *asp* is a genuine gene. First, in a monumental study of approximately 23,000 HIV and SIV sequences, Cassan and colleagues convincingly showed that the ASP ORF is exclusively found in group M viruses and correlates with subtypes, or clades, responsible for the human pandemic [65]. Next, mutations in *asp*, not reflected in overlapping *env* sequences, are associated with CCR5 and CXCR4 tropism [66]. Finally, the effect of natural selection on *asp* as a functional gene was examined. Analysis of the ratio of nonsynonymous to synonymous substitutions per site on both strands of *env* DNA strongly suggests *asp* is being conserved in the HIV-1 genome as a real gene [67]. Taken together, these data provide a strong theoretical case that *asp* not only is a genuine gene, but that the expressed protein may play an important role in HIV-1 pathogenicity.

Experimental evidence of antisense HIV-1 transcription was first published in 1990 with the observation of polyadenylated transcripts 1.0, 1.1, and 1.6 kb in length identified in H9 cells infected with HIV-1 [68]. The transcripts were present early in infection but not on days 5, or later, postinfection. Subsequently, Michael and coworkers confirmed the production of negative-strand transcripts in HIV-1-infected peripheral blood mononuclear cells. Two short transcripts, as well as a longer 2.3 kb transcript, were found transcribed using a novel promoter in the 3′LTR that is downregulated by Tat [69,70]. Other investigators found antisense mRNA transcripts in a variety of HIV-1-expressing cell types [71,72,73,74]. Evidence for spliced antisense transcripts has also been reported [75,76]. In addition to serving as the template for protein translation, these transcripts appear to have a regulatory function by suppressing viral replication and promoting viral latency via complementary binding to the virus sense transcripts, or by acting as a long noncoding RNA that recruits epigenetic and transcriptional silencers to the 5′LTR [76,77,78,79,80,81]. Torresilla and colleagues estimated the size of the antisense transcript at 4.1 kb [82]. Importantly, antisense transcription was preferentially activated in primary monocyte-derived cells [72,73]. Thus, there is strong experimental evidence for expression of antisense mRNA transcripts in the course of HIV-1 infection of susceptible cells [54], and such transcripts may play a role in virus persistence [83].

Convincing experimental evidence exists that HIV-1 produces an antisense protein representing the 10th known virus protein. First, indirect evidence for production of ASP was suggested by the discovery of an immune response directed against the protein during HIV infection of humans. Antibodies against ASP were detected in numerous patient sera [84]. A recently developed luciferase immune-precipitation system was used to quantitate the humoral immune response in HIV-1-infected patients and delineate the epitopes of ASP targeted by antibodies [85]. Also, HIV-1-infected individuals were shown to mount a CD8 T-cell response against ASP [86,87,88]. The protein itself was eventually found in various HIV-1-infected cell lines and in leukocytes [89,90]. The first studies on the possible function of ASP revealed that it induced autophagy [91,92], positively modulated HIV-1 replication in macrophages, and may play a role in immune evasion [82]. Recently, evidence was reported that ASP is a transmembrane protein, residing on the surface of infected cells, and an integral protein in the HIV envelope [93]. Overall, experimental studies confirmed that *asp* is a genuine gene from which a protein is expressed during the course of HIV-1 infection [54].

### 6.4. Additional Smaller Antisense Transcripts and Proteins in HIV-1

While 2.3 kb [71] or 4.1 kb [82] polyadenylated antisense mRNA transcripts encode the ASP of HIV-1, smaller antisense transcripts may also encode proteins. Studies by Ludwig and coworkers have obtained evidence for production of small antisense proteins from such transcripts [71]. A region within the antisense transcripts, termed MORT, forms a microRNA-like structure that may act to induce apoptotic cell death [94]. In addition, the transcripts overlap with all known sense mRNAs, and have the potential for regulating these transcripts via complementary binding [95]. A number of small ORFs are present in these antisense transcripts, but they lack a canonical start signal for protein translation. Thus, translation of the ORFs would need to employ ribosomal frameshifting, noncanonical start codons, or another mechanism. However, there is evidence that these small HIV-1 antisense proteins, termed HAPs, are expressed in HIV-1-positive peripheral blood leukocytes [71]. Unfortunately, the sequence conservation of HAPs is unknown. Additional studies, such as those done for *asp* [65], could assist in determining whether they may, like ASP, may play a role in the HIV-1 pandemic.

## 7. Antisense Transcripts and/or ORFs in the 3′ Genome Region of Other Exogenous Retroviruses and in Endogenous Human Retroviruses

### 7.1. Animal Lentiviruses

Animal lentiviruses have been examined for the presence of antisense ORFs and mRNA transcripts. An antisense ORF has been identified for simian immunodeficiency virus [55,64,65] and Visna virus of sheep. In addition to an antisense ORF, production of antisense transcripts has been identified for both bovine immunodeficiency virus [96] and feline immunodeficiency virus (FIV) [97,98]. Furthermore, preliminary data indicate that an antisense protein is expressed during FIV infection of cats, and an immune response against the protein is generated in the natural host (J. Elder, unpublished data). Thus, animal lentiviruses possess antisense ORFs with mRNA transcripts, and translated proteins, observed during virus replication.

### 7.2. HTLV-1

**Figure 3:**
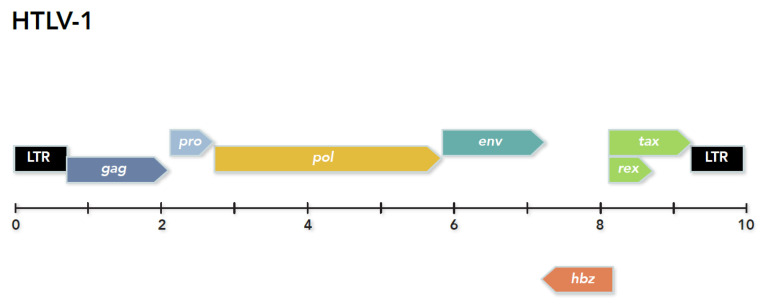
Organization of the HTLV-1 genome.

Antisense transcripts, and translated proteins, have been identified in deltaretroviruses. Based on the prediction of an antisense ORF and transcript in HIV-1, and an antisense ORF in HTLV-1 [55,64], Larocca and colleagues searched for, and found, antisense transcripts in human T cells infected with HTLV-1 [99]. This was the first published evidence for antisense transcripts produced in cells infected with a human retrovirus. The ORF contained within the transcripts was found to have “no extended identity” with the predicted HIV-1 ASP. Subsequently, it was shown that an antisense protein was translated from the transcript. The protein was identified as a bZIP transcription factor that down-regulates viral transcription [100,101]. The protein, termed HTLV-1 bZIP factor, or HBZ, was the first published physical evidence that an antisense protein was produced in retrovirus-infected human cells (Figure 3).

This discovery, in addition to being a major conceptual breakthrough, overturned the hegemonic position of the Tax protein in pathogenesis, discussed above, and opened a new perspective into the oncogenic process related to HTLV-1 infection. First, it was shown that the HBZ protein is mainly found in CD4+ T cells in vivo [101,102,103,104,105]. Next, it was found that HBZ interacts with several cellular transcription factors (i.e., CREB, CREB2, JunD, c-Jun, JunB, and the p65 subunit of the NF-κB complex) and modulates their activation, thereby influencing the expression of a large number of both host gene and viral genes. Thus, HBZ inhibits HTLV-1 sense transcription by recruiting essential transcription factors, and also affects many other cellular processes including host gene expression, innate immune signaling, apoptosis, autophagy, and DNA repair, all of which have an important influence on the pathology of HTLV-1 infection [54,101,103,105]. The involvement of HBZ in the development of ATL offered new insights into its role in the regulation of signaling cascades and the expression of genes involved in cell cycle control [101]. Although some studies imply that HBZ is not essential for HTLV-1-induced immortalization, HBZ has been shown to positively affect T-cell proliferation [106], and that transgenic mice expressing HBZ developed T-cell lymphoma [102]. Thus, the antisense protein HBZ plays a crucial role in the pathogenesis of HTLV-1 [101,103,105].

### 7.3. Other Human and Animal Deltaretroviruses

Other human, as well as animal deltaretroviruses, have been examined for the presence of antisense ORFs and transcripts. An antisense ORF was identified in bovine leukemia virus (BLV) and HTLV-2 genomes [55]. Spliced antisense transcripts have been identified in the human deltaretroviruses HTLV-2, -3, and -4; simian counterparts STLV-1, -2, -3, and -4 [107]; and antisense proteins found to be translated. In the case of BLV, a transcript is produced [96,108], but there is no evidence that it is translated. Among those mentioned, the antisense protein of HTLV-2, termed APH-2, has been well studied [109]. APH-2 is 183 amino acids long and is localized in the cell nucleus. It lacks a consensus basic leucine zipper domain, and thus does not function as HBZ in HTLV-1. While HBZ and APH-2 both repress Tax2-mediated transcription, they modulate the virus and cellular pathways differently [110,111]. This may explain why HTLV-2 does not generally cause disease. Overall, antisense transcripts, and proteins translated from these mRNAs, represent a common feature of both human and animal deltaretroviruses’ replication and gene expression. On the other hand, although the HIV ASP and HTLV-1 HBZ proteins possess different characteristics, they appear to have a common function in modulating the immune response and viral gene expression, and likely contribute to chronicity of infection [101].

### 7.4. Gammaretroviruses

Little is known about the presence of conserved antisense ORFs, or expression of antisense transcripts, in members of the Gammaretrovirus family. However, there is evidence for antisense transcription for one member. Rasmussen and coworkers found transcriptional activity within the U3 region of the 5’LTR of proviral murine leukemia virus (MLV; [112]). This could represent a mechanism of insertional activation of host genes. Interestingly, it is known that HBV is most like MLV among all retroviruses examined [55,63,113]. Hence, it is possible that antisense ORFs, and expressed proteins, will eventually be identified in gammaretroviruses.

### 7.5. Endogenous Human Retroviruses

Human endogenous retroviruses (HERVs) represent an abundant class of repetitive retroelements in the genome of *Homo sapiens* [114], comprising approximately 8% of the human genome. Among the best studied are endogenous retrovirus 9 (ERV-9) and human endogenous retrovirus-K (hERV-K). About 50 copies of ERV-9 are integrated per cell. Both sense and antisense transcripts specific for ERV-9 are produced in malignant and nonmalignant human cells. While antisense transcripts are expressed at the same level as sense transcripts in malignant cells, they are found in higher levels in nonmalignant cells. Deregulation of the antisense transcript is thought to promote tumor formation [115]. In addition, the long terminal repeats (LTRs) of hERV-K have been shown to contain bidirectional promoter activity [116,117]. Thus, both sense and antisense transcripts are produced by these endogenous retroviruses. Whether proteins are translated from the antisense transcripts remains to be determined.

### 7.6. Hepadnaviruses

After identification of conserved antisense ORFs in hepadnaviruses, a search for an antisense transcript, and expressed ORF6 protein, was undertaken experimentally in woodchucks infected with WHV. Examination of the livers of woodchucks acutely or chronically infected with the virus yielded negative results when searching either for the antisense transcript or ORF6 protein [118]. However, a cell-based transcription assay did find an antisense transcript expressed by WHV [119]. Similar experiments on HBV-infected human cells also identified the presence of an antisense transcript [120]. Finally, an HBV antisense transcript of 0.7 kb, which encompasses ORF6, was found in liver tissue of humans infected with HBV [121]. Interestingly, the latter transcript is not polyadenylated, and is thought to be produced by RNA polymerase III. Overall, there is evidence that an antisense transcript is produced during the course of hepadnavirus gene expression. While ORF6 is conserved in hepadnavirus genomes, and a corresponding antisense transcript documented, there is no experimental evidence that an antisense protein is produced [58]. This could be due to a number of reasons: (1) perhaps ORF6 is no longer a genuine gene; (2) an antisense protein could be expressed, but in amounts below detection levels; or (3) a protein is expressed but is quickly degraded or masked from detection. If expressed, it appears unlikely that the ORF6 protein plays an essential role in WHV replication. Site-directed mutagenesis, to introduce premature termination codons in the WHV ORF6 coding sequence, yielded a virus that produced an infection in woodchucks indistinguishable from infection with wild-type virus, and the result was not due to reversion of the mutations [61]. Thus, it is possible that ORF6, in the WHV genome, represents a vestigial gene. However, the role of the ORF6 protein in HBV replication, or in other hepadnaviruses, remains unanswered.

The fact that the antisense ORF6 transcript is not polyadenylated would lead one to speculate that a protein is not produced. However, it has been shown that human cells can translate proteins from such transcripts [122]. So, it is possible that the ORF6 protein is expressed in low levels during replication of some hepadnaviruses. Overall, clear evidence exists that an antisense transcript is produced during the course of hepadnavirus infection. However, it is not known what role, if any, this transcript plays in virus replication and/or in gene regulation or expression. Overall, antisense transcription appears to be a common feature for human and animal retroviruses, retrovirus-like viruses (e.g., hepadnaviruses), and even human endogenous retroviral elements.

## 8. Barriers to Overcome in the Study of HIV-1 ASP: From Technical Difficulties to Epistemological Blocks

### 8.1. Technical Difficulties

Detection of ASP is particularly difficult. First, specific antibodies against ASP are difficult to produce due to challenges in expressing recombinant ASP. Development of several tools was needed early in the study of the protein. Expression of ASP in eukaryotic cells was first accomplished using the insect Sf9 cell line overexpressing ASP via codon optimization of its cDNA [91], followed by exploiting the properties of a favorable expression vector, p-CAGGS [85,123]. Even when present in infected cells, ASP is difficult to detect due to its physical characteristics (e.g., hydrophobicity), multimerization properties, potential binding to other proteins or nucleic acids, or its rapid degradation in the host cell. What future approaches could be employed to demonstrate the role of ASP, and its transcript, in HIV-1 pathogenesis? First, more sensitive and specific detection methods should be developed and deployed. Second, studies should be conducted to optimize the likelihood of detection (e.g., time course of natural infection in humans and human cells, experimental investigations in animal models, etc.). Third, further development of appropriate animal models should be an important focus. Such approaches may help demonstrate the role of antisense in the replication cycle and pathogenesis of HIV-1.

### 8.2. Epistemological Blocks

Doubt, but above all skepticism and disbelief, has prevailed within the scientific community throughout the major stages in the history of virology, and of retrovirology, in particular. Of course, methodological skepticism is part of the scientific approach in the testing of hypotheses. However, excessive skepticism can lead to fixism. While the importance of the HBZ protein and transcript in HTLV-1 replication, gene expression and pathogenesis is firmly established, many in the field seem reluctant to consider that antisense transcripts and proteins play a comparable role in the biology of HIV-1. Several explanations are possible. First is the disciplinary organization of science that can stifle new ideas. This was instituted in the 19th century, and more fully developed in the 20th century, and is particularly evident in the complex field of biology. Certainly, disciplinary excellence is essential to ensure the quality of scientific research. However, hyperspecialization can be a powerful brake on scientific curiosity. In the case of HIV-1, researchers have often neglected knowledge of related viruses (i.e., hepadnaviruses), as well as other retroviruses (e.g., deltaretroviruses). In consequence, HIV-1 investigators may not be aware of the work demonstrating the crucial importance of the antisense protein HBZ in HTLV-1 pathogenesis. Another epistemological block may be linked to the idea that the irreversible loss of genes as time goes on is a characteristic of all parasitic life forms (a concept of “reductive genome evolution”). This concept is particularly well documented for endosymbiotic/parasitic bacteria such as mitochondria [124]. However, viruses, which are parasites of their cellular host, seem not to follow this phenomenon of genome reduction. For example, DNA viruses and some RNA viruses (e.g., *Retroviridae*), were thought efficient in capturing both cellular genes, as well as viral genes from other family viruses, gaining functions and genes over time (de novo gene creation), rather than losing them. Although it was considered that the de novo creation of genes was a very rare event, recent evidence suggests that de novo creation of genes occurs frequently, particularly in viruses, either in intergenic regions and introns or in genomic regions that contain a gene (i.e., overprinting). HIV-1 is a complex retrovirus that has evolved throughout its history. Therefore, it should not be surprising that in the course of its evolution the creation of a new gene (i.e., *asp*) could occur [125]. Finally, probably the most important barrier for acceptance is the lack of a clear function(s) for ASP. Thus, although significant theoretical evidence is available, at the current time there is no experimental evidence directly linking ASP to viral pathogenesis, and the protein remains difficult to detect in infected cells. Skepticism, without consideration of all relevant facts, is unproductive. Perhaps we are in a situation described by Khun in the “structure of scientific revolutions”, in a period of paradigm shift resulting from anomalies or daring questions that run counter to “normal” science, “when scientists can no longer ignore anomalies that overturn the established situation in scientific practice, then begin the extraordinary investigations that finally lead them to a new set of beliefs, on a new basis for the practice of science” [126]. After a period of “crisis,” a certain amount of time is needed for members of the scientific community to accept the new viewpoint. Thus, once the function, or functions, for ASP are established, this epistemological tension will be largely resolved.

## 9. Summary and Conclusions

Thirty-three years after first predicted, antisense mRNA transcripts, and in many cases translated proteins, are known to be common for many exogenous retroviruses, retrovirus-like viruses, and endogenous retroviruses. The antisense ORFs are relatively small, potentially encoding proteins less than 200 amino acids and found in the 3′ region of the linear viral genome. This region is organizationally complex possessing both overlapping genes as well as noncoding signal sequences inside of genes (e.g., RRE within *env*). Due to their widespread conservation, it is reasonable to postulate those antisense transcripts, and their translated proteins, play a role in virus replication, gene expression, and/or pathogenesis. It is clear that the antisense transcript and protein of HTLV-1 significantly contribute to virus pathogenesis in humans. However, the role of antisense proteins, and transcripts, in HIV-1 infection remains to be determined. Many questions persist. First, are antisense genes a recent addition to the retrovirus genome as suggested by Cassan and colleagues and Gholizadeh and colleagues [70,125], or were they inherited from the ancestor of these viruses? Second, since antisense transcripts are complementary to the virus sense transcripts, do they play a role in transcription control and/or latency via competitive binding, or other mechanisms? Third, does the antisense protein of HIV-1 play a role in pathogenesis? Finally, since some retroviruses appear to have vestigial antisense genes, have other virus proteins evolved to assume the function of defunct antisense proteins? Currently, there are more questions than answers in this area of investigation. Clearly, definitive studies are needed to determine the function of ASP. The fact that HBZ plays an essential role in HTLV-1 pathogenesis, coupled with the finding that *asp* has all the characteristics of a genuine gene, and has been maintained solely in virus clades involved in the HIV-1 pandemic, speaks to an important, if not essential, role of ASP in pathogenesis. Since sterilizing immunity has not been achieved by any interventional strategy yet employed to combat the AIDS epidemic, we suggest that ASP, and its mRNA transcript(s), be explored as potential targets in anti-HIV therapeutic and vaccine strategies.
